# Barriers and enablers to a physician-delivered educational initiative to reduce low-acuity visits to the pediatric emergency department

**DOI:** 10.1371/journal.pone.0198181

**Published:** 2018-05-29

**Authors:** Gregory Huyer, Samia Chreim, Wojtek Michalowski, Ken J. Farion

**Affiliations:** 1 Telfer School of Management, University of Ottawa, Ottawa, Ontario, Canada; 2 Departments of Pediatrics and Emergency Medicine, University of Ottawa, Ottawa, Ontario, Canada; 3 Emergency Department, Children’s Hospital of Eastern Ontario, Ottawa, Ontario, Canada; Donders Institute for Brain, Cognition and Behaviour, NETHERLANDS

## Abstract

**Background:**

Use of the pediatric emergency department (PED) for low-acuity health issues is a growing problem, contributing to overcrowding, longer waits and higher health system costs. This study examines an educational initiative aimed at reducing low-acuity PED visits. The initiative, implemented at an academic pediatric hospital, saw PED physicians share a pamphlet with caregivers to educate them about appropriate PED use and alternatives. Despite early impacts, the initiative was not sustained. This study analyzes the barriers and enablers to physician participation in the initiative, and offers strategies to improve implementation and sustainability of similar future initiatives.

**Methods:**

Forty-two PED physicians were invited to participate in a semi-structured individual interview assessing their views about low-acuity visits, their pamphlet use, barriers and enablers to pamphlet use, and the initiative’s potential for reducing low-acuity visits. Suggestions were solicited for improving the initiative and reducing low-acuity visits. Constant comparative method was used during analysis. Codes were developed inductively and iteratively, then grouped according to the Theoretical Domains Framework (TDF). Efforts to ensure study credibility included seeking participant feedback on the findings.

**Results:**

Twenty-three PED physicians were interviewed (55%). Barriers and enablers for pamphlet use were identified and grouped according to five of the 14 TDF domains: social/professional role and identity; beliefs about consequences; environmental context and resources; social influences; and emotions.

**Conclusions:**

The TDF provided an effective approach to identify the key elements influencing physician participation in the educational initiative. This information will help inform behavior change interventions to improve the implementation of similar future initiatives that involve physicians as the primary educators of caregivers.

## Introduction

Hospital emergency departments (EDs) are highly specialized environments designed to treat acute illnesses and injuries requiring immediate attention. However, many ED visits are in fact for non-emergent or low-acuity conditions, representing 48% of visits to Canadian EDs in 2010–11 [1]. The situation is similar in pediatric EDs (PEDs); for example, 55% of PED visits in the province of Ontario, Canada in 2005–06 were classified as low acuity (defined as triage levels IV or V on the five-level Pediatric Canadian Triage and Acuity Scale) [2]. The problems created by low-acuity use of the PED have been extensively studied and documented [3–6]. Perhaps the most significant issue is overcrowding, leading to increased wait times for those who actually require emergent medical attention. Low-acuity use of the PED also leads to increased health system costs, as an ED visit is significantly more expensive than being seen by a primary care provider [7–9]. In fact, estimates suggest that over $4 billion annually could be saved in the United States if all non-emergent care was diverted from the ED to retail clinics [9].

A key goal of all publicly-funded health care systems is to deliver evidence-based care in the most cost-effective setting while achieving high-quality outcomes. To this end, diverting low-acuity patients from the PED to primary care in the community is highly desirable. Lack of access to primary care is an obvious barrier to reducing low-acuity PED visits; however, Farion *et al*. [10] showed that even among families with primary care providers, visits to the PED were common for low-acuity health problems, as families over-estimated the seriousness of their child’s condition, sought immediate or convenient answers rather than waiting for an appointment with their provider, or believed their child would require investigations or consultations with specialists immediately available in the hospital setting. Other studies have enumerated a variety of similar reasons for low-acuity use of the PED including convenience, medical expertise, perceptions of quality of care, efficiency, and referral from primary care providers [11–15]. Health literacy is also an important determinant, as caregivers may have difficulty distinguishing a low-acuity problem from a true emergency and therefore use the PED for non-emergent conditions [14,16,17].

Researchers and health care providers have developed and implemented a variety of caregiver educational initiatives aimed at reducing non-emergent PED use. Approaches included information booklets or other printed materials, videos and/or educational activities to improve parents’ health literacy about common childhood ailments [17–22], and handouts or counseling on how to obtain medical advice [23,24]. While most programs had some impact on reducing non-emergent PED use, health literacy interventions that relied on a single education and training session tended not to be successful [19,20]. Interestingly, PED physicians were rarely, if ever, involved in delivering these initiatives, even though physician-delivered messages can be very effective in influencing patient behavior [25]. This omission represents a lost opportunity to increase the effectiveness of educational initiatives, as the physician’s knowledge and authority, coupled with his/her established relationship with the patient, suggest that educational messages from physicians carry weight and may be more likely to be retained.

This study examines the implementation and sustainability of an educational initiative—aimed at reducing low-acuity PED visits—that was delivered by PED physicians in a major pediatric academic hospital in Ontario, Canada. As compared to other educational initiatives with similar aims [17–24], this initiative was, to the best of our knowledge, unique in employing physicians as the primary educators. The initiative consisted of physicians discussing the appropriate use of the PED with children’s caregivers at the end of their visit and sharing with them a two-page pamphlet that rated the acuity of their child’s condition, provided information on how to distinguish an emergency from a non-emergency, and identified alternative treatment options available in the community. While physician participation in the initiative was initially strong, pamphlet usage gradually dropped off and within four months the initiative had effectively ceased. We sought to understand the perspectives and behaviors of participating physicians towards the initiative and why it failed to be sustained. Through our qualitative analysis, we also sought to identify the barriers and enablers to PED physician participation and propose ways to improve implementation and uptake of this and similar initiatives. Physicians can play a central role in initiatives intended to educate patients and caregivers; thus, their views and experiences represent valuable information for those making decisions [26] about these types of initiatives.

## Background

The educational initiative examined in this study was developed at the PED of the Children’s Hospital of Eastern Ontario (CHEO), an academic, tertiary PED serving patients up to 17 years of age (with 72,142 patient arrivals in 2016–17). The hospital is located in Ottawa, Ontario, which is bordered by the province of Quebec. CHEO and its PED are the only pediatric centre to serve the large geographic area of eastern Ontario and western Quebec with a pediatric population of 500,000.

The initiative was implemented in May, 2015, in response to increasing volumes and growing proportion of low-acuity PED visits (S1 Fig). The goal of the initiative was to educate caregivers and influence future PED use by equipping them to better distinguish a true emergency from a non-emergent condition and offer alternative sources for care. The educational initiative included a two-page pamphlet entitled “Choosing Wisely” (S1 File) [27] that consisted of four sections: (1) explanation of the importance of appropriate PED usage; (2) rating of the seriousness of the presenting child’s condition; (3) examples of emergency and non-emergency conditions; and (4) alternatives to PED in the community and resources for finding a primary care provider. (The name “Choosing Wisely” was meant to reflect the goal of having caregivers “choose wisely” when deciding to bring their child to the PED, rather than indicate a connection with the Choosing Wisely movement which seeks to educate physicians and patients on avoiding unnecessary medical tests, treatments and procedures.) The pamphlet was developed by a multidisciplinary team including PED physicians, hospital administrators, public relations and patient education experts, with input from and piloting by parent groups. The pamphlet’s design and content were based on published initiatives aimed at educating patients and caregivers about ED usage [18–22,24] and other educational pamphlets developed at CHEO. Initiatives that were successful in reducing PED visits (for example, [18,21–24]) typically involved multiple contacts with the caregivers and/or provided personalized and targeted information. Thus, there were intentions to couple this “Choosing Wisely” initiative with other efforts, including a public education campaign in partnership with local public health officials and primary care physicians, and advocacy for improved primary care access, but these were not fully implemented. Consistent with best practices, the pamphlet was written at an accessible literacy level; in addition, the pamphlet was available in English and French to meet the linguistic needs of the vast majority of patients and caregivers cared for at CHEO.

A unique aspect of this initiative was the use of PED physicians to provide the educational message, as opposed to nurses, trained patient educators, and/or research staff. PED physicians were asked to discuss the pamphlet with caregivers at the end of the visit, just before the patient’s discharge. The structure of the discussion followed the organization of the pamphlet. The discussion was expected to be brief—five minutes or less. The pamphlet was intended to be given to the caregivers of all patients, with the exception of those with seriously ill children and/or situations where there was conflict or a high potential for conflict. The physicians were provided instructions and training on using the pamphlet, including speaking notes and responses to common questions to help guide their discussions. One week after implementing the initiative at CHEO, a local media release was conducted to sensitize the public to the initiative. This media release received extensive local coverage, as well as some national interest. The pamphlets were initially left in the PED for physicians to take and distribute on their own but were later attached to patients’ paper charts by PED clerks to facilitate and promote usage.

The weekly average of daily patient arrivals to the PED before and after implementation of the initiative was tracked and compared with the two previous years (S2 Fig). In the 10 weeks prior to the initiative launch, a sustained period of high PED utilization was observed, over-and-above that for 2013 and 2014. However, after the initiative was implemented, a decline in PED utilization occurred, over-and-above the typical seasonal decline associated with the summer period. A confounding factor that may have contributed to the decline was the opening of an urgent-care walk-in clinic for pediatric cases in nearby Gatineau, Quebec, which may have diverted some patients from Quebec who would have otherwise visited the PED at CHEO. In the Fall when the initiative had effectively been discontinued, the PED utilization rates returned to the levels typical of previous years. These changes in the pattern of PED utilization suggested that the initiative may have had an effect in reducing low-acuity PED visits, although we cannot say with certainty whether the relationship was causal or simply correlative.

## Methods

### Ethics

The study was approved by the CHEO Research Ethics Board (protocol no. 16/35X). Participation was voluntary, and each eligible physician’s response to recruitment was managed through a third party to avoid an authority gradient from their colleague investigator (KF, recent division chief). Similarly, interview transcripts were anonymized with a study ID number and only coded summaries were available to the physician investigator (KF).

### Participants

PED physicians who worked in the PED at CHEO in May/June 2015 when the initiative was launched and who were still on staff in the summer of 2016 when data collection began were eligible to take part in the study. Forty-two physicians met the inclusion criteria and were invited to be interviewed.

### Interviews

Interviews followed a semi-structured format with probes. Physicians were first asked about their understanding and beliefs about low-acuity PED visits. Next, they were asked whether and how they had used the Choosing Wisely pamphlet, followed by exploration of the barriers and enablers of using the pamphlet and their views on the initiative’s potential for reducing low-acuity PED visits. The interview also included questions about participants’ views of how the educational initiative could be improved and what other steps could be taken to reduce low-acuity PED visits.

### Analysis

All interviews were digitally recorded and transcribed verbatim. ATLAS.ti version 7.5 was used to facilitate data coding and retrieval of coded quotations. We used constant comparison in the analysis, adopting an inductive approach [28,29]. In the early stage of the analysis process, two researchers (GH and SC) independently coded one interview, and then jointly reviewed and discussed the codes to develop a preliminary code list. As a second step, the first author (GH) used the preliminary list to code additional interviews. The process of coding was iterative [28,30] and involved “moving back and forth within and between transcripts” to identify and then validate codes (see page 20 in [31]). As additional interviews were coded, the code list was refined by the two researchers (GH and SC) to incorporate emerging themes. For example, initially in the analysis, we used a single code for “how the physician used the pamphlet”. However, as more interviews were analyzed, it became apparent that the time required to deliver the message was an important factor. Thus, the code was replaced with more specific codes to capture how often the physician used the pamphlet, how the physician delivered the message, and the time required to deliver the message. The first author (GH) then recoded all interviews using the revised code list.

After the initial coding was completed, we grouped the identified barriers and enablers into categories following the Theoretical Domains Framework (TDF) [32]. The TDF was chosen as it was designed and has been validated to investigate and describe problems associated with implementing new practices [33]; moreover, our initial codes and categories aligned well with a number of the TDF domains. The initial mapping of factors to TDF domains was done by the first author (GH) and independently validated by the other authors to arrive at a consensus.

### Ensuring quality of findings

We followed the Consolidated Criteria for Reporting Qualitative Research (COREQ) [34] to ensure the quality of the study, which included researcher triangulation in coding, reporting extensive quotes from the interviews, and conducting participant checks. All physicians who were interviewed and still on staff were sent a copy of the draft manuscript and asked to confirm whether they agreed with the findings. Nearly 60% of the contacted physicians responded, and all expressed their agreement with the analysis and conclusions. Three physicians offered minor suggestions on the text that were incorporated into the final version submitted for publication.

## Results

Of the 42 physicians who met the inclusion criteria, 23 agreed to be interviewed (55%). Interviews lasted on average 40 minutes. The physicians’ years of experience at the hospital ranged from 1.5 to 31 years (mean of 12 years, median of 8 years).

All physicians noted that low-acuity visits represented a significant proportion of PED visits. Physicians generally agreed that low-acuity visits were problematic and needed to be reduced, citing a variety of consequences including overcrowding, longer wait times, the risk of not detecting an incorrectly triaged high-acuity patient, and general staff frustration and stress. Regarding the potential risks of the pamphlet’s message being misinterpreted, physicians noted the possibility of some caregivers being deterred from bringing their children to the PED with a true emergency in the future, but in general they thought this would be unlikely and that the benefits of the initiative outweighed the risks.

While all physicians interviewed had used the pamphlet, their participation in the initiative was inconsistent. Only about one quarter of the physicians gave the pamphlet to all caregivers regardless of the acuity of the child’s condition, as intended. The majority of physicians targeted the pamphlet only to low-acuity visits, with some also using the pamphlet for medium-acuity visits as a way to reinforce that those caregivers had made the correct choice in coming to the PED. In terms of the discussion with caregivers, physicians typically reviewed the entire pamphlet, although the section rating the present visit was sometimes omitted. In all cases, the physicians discontinued handing out the pamphlet over the course of a few months.

**Fig 1:**
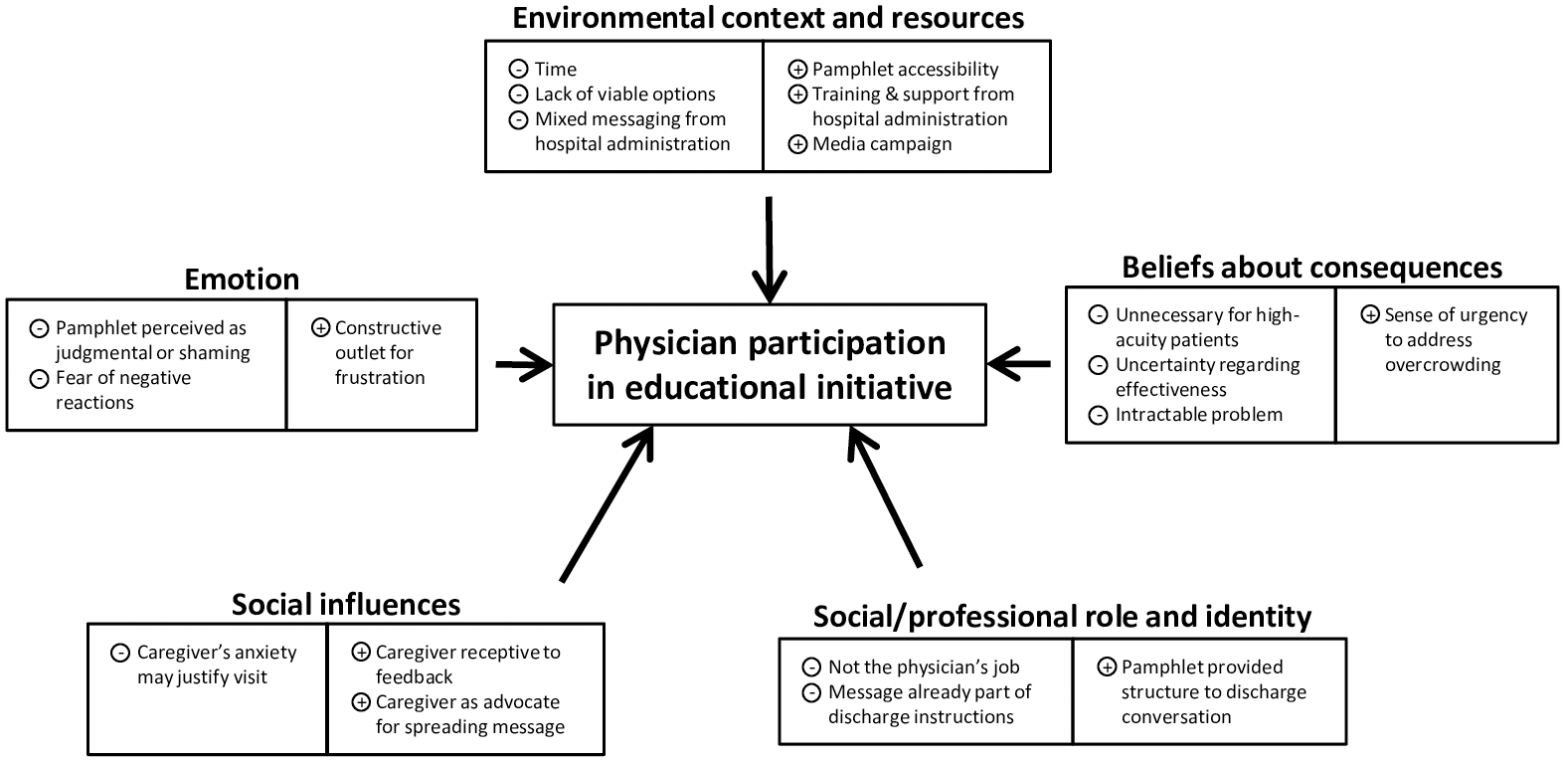
Model summarizing key TDF domains influencing physician participation in the initiative. The five TDF domains are shown with the barriers and enablers that map to each domain listed below the name of the domain. Barriers are indicated with a “-” sign and enablers with a “+” sign.

Analysis of the interview coding revealed a number of barriers and enablers that influenced physician participation in the initiative. The factors were grouped according to the TDF domains; of the 14 domains in the framework, five were found to encompass all the barriers and enablers (Table 1) and are elaborated in more detail below. In addition, a model summarizing the domains and influences associated with physician delivery of the educational message is shown in Fig 1.

**Table 1:** Thematic grouping of barriers and enablers for physician participation following the domains of the TDF.

TDF Domain	Barriers	Enablers
**Social / professional role and identity**	Message already part of physician discharge instructions Not the PED physician’s job to deliver message	Pamphlet provided more structure to discharge conversation
**Beliefs about Consequences**	Message seemed unnecessary and inappropriate for high-acuity patients Uncertainty regarding effectiveness of the initiative Changing caregivers’ behavior seen as an intractable problem	Sense of urgency regarding need to address PED overcrowding
**Environmental context and resources**	Time required to discuss pamphlet Mixed messaging from hospital administration about pamphlet usage Lack of viable options to the PED in the community	Pamphlet accessibility (attached to chart) Training and support from hospital administration Media campaign to promote awareness of initiative
**Social influences**	Caregivers’ anxiety may justify the visit, even if the child’s condition doesn’t	Caregivers who asked for or were receptive to feedback about the appropriateness of their visit Caregivers who had come appropriately and could be recruited as advocates to spread the Choosing Wisely message
**Emotion**	Physician perceived pamphlet as judgmental or shaming Fear of negative reactions from caregivers	Constructive outlet for expressing frustration over unnecessary visits

### 1. Social / Professional role and identity

This domain refers to the behaviors and qualities of an individual in a social or work setting, and whether a change in behavior is seen as being compatible with one’s professional role and identity. The barriers and enablers that mapped to this domain all related to the way in which the initiative fit with how the physicians perceived and carried out their role. In terms of barriers, some physicians, especially those with more years of PED experience, noted that they routinely discuss appropriate PED use with the caregivers as part of their role, which involved providing discharge instructions. As such, they found it redundant to use the Choosing Wisely pamphlet. For example, a physician stated:

*I have a shortened version [of the message] without actually handing them a piece of paper about where they should be seeking care*.

[Physician 19]

In addition, some physicians questioned whether other aspects of their role (most notably patient care) should be given a higher priority than educating caregivers, and that perhaps other staff such as nurses or a dedicated patient educator could also take on the role of caregiver educator.

*Am I the best person when I’ve just treated their child? I’m not convinced. Am I the best person when it’s a busy department, and this is taking up time that I could be using to teach residents and fellows, seeing the next patient which is what I’m here for? … Yes, I may have the most understanding of how sick you are today but I think a skilled nurse or a retired nurse or someone who can do a pretty good job of figuring out who needs to be here and who doesn’t and can work along with other educational pieces*.

[Physician 9]

In terms of enabling factors, participants with fewer years of experience in the PED physician role were more likely to report that the pamphlet was helpful in terms of giving more structure to their discharge instructions. They appreciated having a tangible document that they could refer to and that could facilitate their role of educating and communicating with caregivers.

*It made me be a little bit more specific with parents as to what they needed to do, with regards to their current presentation, where they could go next time in situations like this. … I definitely prefaced my discharge instructions with that explanation, which I don’t normally, and then a little bit more details in terms of specifics around what can be provided by a primary care doctor*.

[Physician 2]

### 2. Beliefs about consequences

This domain is associated with acceptance of the truth, reality or validity about outcomes of a behavior in a given situation, capturing constructs related to beliefs and outcome expectancies. The barriers and enablers relevant to this domain related to physicians’ belief in the need to address overcrowding versus their belief in the utility and effectiveness of the “Choosing Wisely” initiative in changing caregiver behavior. An important enabler was the fact that almost all physicians noted that the high volume of low-acuity visits created problems for patients, families and staff, and that it was important to do something about it. This belief about the need to decrease volume was a motivating factor in encouraging physician participation in the initiative.

*An enabler could be the physician who is fed up with the volume, that sort of motivation. When it’s really terrible, I think we’re willing to try anything*.

[Physician 14]

However, several factors related to beliefs about consequences served as barriers to physician participation. First was the sense that the message was unnecessary and inappropriate for high-acuity cases. As noted above, the pamphlet was intended to be used for all patients. However, the majority of physicians felt that it was awkward and inappropriate to deliver the message to caregivers who had come for an emergent condition, and instead they targeted the message only to low-acuity visits.

*For the kids who really needed to be there, … it was awkward to show this pamphlet because they came for the right reason. To say, “You’re here today; yup, your child had a true emergency”, it seemed like a redundant conversation: it didn’t really seem like it was the right conversation to be having with those parents*.

[Physician 17]

Second, physicians questioned the efficacy of the initiative and said they would have been more inclined to use the pamphlet if they believed it was having an effect on the number of low-acuity visits. Physicians noticed a brief drop in PED visits after the launch of the pamphlet, but they did not know if it was due to the Choosing Wisely initiative or to other factors, such as the associated media attention or the opening of a pediatric urgent-care clinic in Quebec. Some were also critical of the lack of empirical evaluation of effectiveness (for example, pre-/post-test measures of number of low-acuity visits).

*If I felt like it was making a difference and people felt that it was helpful to them, I think I would be much more likely to use it*.

[Physician 23]

Finally, a significant barrier to physician’s use of the pamphlet was the belief that changing caregivers’ behavior was an intractable problem from a broader societal perspective. Notably, this barrier was cited more commonly by physicians with a greater level of experience. While the physicians viewed the educational information contained in the pamphlet as being helpful, they felt that a two-page pamphlet would be insufficient to influence behavior in a sustained manner.

*I just don’t know that a piece of paper handed to you after your child has received care, is going to do anything to change behavior. Behavior is so complex…, there are so many different reasons why a person comes, I felt like it was a bit futile*.

[Physician 14]

### 3. Environmental context and resources

This domain captures circumstances of a person’s situation or environment that discourages or encourages the behavior, including material resources, availability of time, and organizational culture. These barriers and enablers included practical logistics related to pamphlet use, factors related to the hospital administration, and broader health system issues. First, at the micro level, a barrier that physicians commonly cited was the time required to discuss the pamphlet with caregivers. Though the time physicians spent was fairly short (typically less than five minutes), they were concerned that the cumulative time over the course of a shift could translate into seeing fewer patients, particularly during periods of higher demand.

*One of the big things for me was, especially on a busy shift, it added anywhere from 3 to 5 minutes to a conversation, and that for 20 to 30 patients adds up and then it cuts down the patients that you can actually see*.

[Physician 2]

Related to the importance of the time required to deliver the message, a key enabler of using the pamphlet was having it attached to the patients’ charts or otherwise easily accessible. Physicians were much more likely to use the pamphlet if it was attached to the charts than if they had to remember to pick up and distribute the pamphlets on their own.

*Having it visible on every chart—that’s the only way you’re going to do it. Because we’re never going to have more time. … Just having it available, that’s what helped me*.

[Physician 16]

At an organizational level, a barrier reported by a few physicians was mixed messaging from the hospital administration about using the pamphlet, which created confusion and contributed to why they discontinued using it. The physicians noted that the administration was strongly supportive of the initiative when it was launched. However, not long after, PED visits decreased, which according to the physicians caused the hospital administration to become concerned about decreased revenue. While the physicians said there was no explicit instruction from the administration to stop using the pamphlet, they nonetheless believed the administration at that point wanted them to avoid discouraging people from coming to the PED.

*I’m feeling kind of mixed about it now because I don’t really understand the messaging that’s being given in terms of whether or not I should be using it. I’d say that’s the biggest barrier, because I’m not really sure where we’re going with it*.

[Physician 3]

An enabling factor related to organizational culture was training and support provided by the hospital administration regarding pamphlet use. Physicians with less PED experience (below the median years of experience) were more likely to report that this training helped them use the pamphlet effectively.

*There had been some education for us from the chief before piloting this tool: we were told how to use it in terms of the script for using it. Maybe that’s the reason [that I had no negative reactions to the pamphlet]*.

[Physician 12]

Interestingly, another enabler was the media campaign after the initiative began to create public awareness. The resultant publicity was found to be helpful as it sensitized caregivers to the pamphlet’s message, with some caregivers even asking about it before the physician mentioned it.

*For me, it made it easier to tell families, “You may have already heard of this”, which made that part of the discussion a lot easier*.

[Physician 10]

Finally, an important macro-level barrier was that physicians found it difficult to discourage caregivers from bringing their child to the PED for non-emergent issues because of lack of viable options for quality primary care in the community, particularly outside of regular working hours due to resource constraints at a systems level. Indeed, for families in which both parents work and have limited time off for work, the PED may be the only practical option.

*You can say “call your family doctor or pediatrician” but if they don’t have a family doctor or pediatrician, that goes away. If it’s after hours, they may not have access… If there are really no other options, or very limited other options, what can you tell them? … I can’t fix the problem that mom and dad both need to work and can’t take a day off work. So they’re going to come here Sunday at 8 pm, irrespective of it because that’s the decision they’ve made that it’s worth that investment of time*.

[Physician 9]

### 4. Social influences

Social influences encompass interpersonal processes such as social pressure, group norms and social comparisons that can cause individuals to change their thoughts, feelings or behaviors. An important influence on physicians, particularly those with a greater level of experience, was the caregiver’s anxiety level. Even though the child’s condition may not be urgent, the physicians believed that the PED visit may be appropriate and necessary to reassure the caregivers, to calm their fears and to improve their understanding of childhood conditions. These physicians were more likely to question the assertion that all low-acuity visits should be diverted to settings other than the PED, and they were therefore less likely to use the pamphlet with caregivers.

*When you’re in front of the individual parent who’s really worried about their child, even if it was crazy that they were worried, … you sort of look into their eyes and think “I get it”*.

[Physician 7]

*No matter how benign their condition, there’s always a level of anxiety above the problem which involves a lack of knowledge and those patients need to be seen. It’s not an unnecessary visit in the sense that those families are experiencing concern about real consequences to their kids, by far most of the time. … So if it’s just providing knowledge I don’t think that’s an unnecessary visit*.

[Physician 15]

On the other hand, an enabler for using the pamphlet was caregivers who were open to or asked for feedback regarding their PED visit. In some cases, caregivers would ask the physician if they needed to come to the PED, or they would even apologize for not having checked with their family physician first. Those cases provided a perfect segue into introducing the pamphlet, and physicians found it helpful to be able to give the pamphlet to caregivers and structure their conversation around it.

*One of the first times I handed it out was a kid I saw on a Saturday morning and precisely that sort of situation where “we would have normally gone to our pediatrician” … I explained [the pamphlet] to them, and they said “yup, we completely understand and we would do what we can in the future to avoid it.” That’s an easy one: it’s a lay-up*.

[Physician 9]

Physicians also found it easier to use the pamphlet with caregivers who had come to the PED for true emergent conditions, allowing them to focus on recruiting those caregivers as advocates to spread the Choosing Wisely message to their friends and family. Part of the pitch to caregivers was that, if there were fewer low-acuity visits to the PED, then children like theirs could be seen by a physician more quickly. Delivering the message this way eliminated any potential for judging or shaming the caregivers since the message about low-acuity PED use was being directed at “others” and not them.

*For the patients who truly, truly needed emergency care, I would give the parents this pamphlet [and] use them as a champion for us. I would say “your child truly needed to be here today, you know how long you waited… So, feel free to take this home and if you have any friends or anyone who’s interested in knowing when a child should be brought to the ED, can you help us educate other people?” That’s when I found the parents to be most receptive, … it was like they felt they were sent on a mission and they felt they were helping out their own child by doing that*.

[Physician 5]

### 5. Emotion

This domain refers to the emotional response or reaction associated with a particular behavior. The initiative provoked significant emotions among physicians that influenced their use of the pamphlet. First, a majority of physicians felt that the pamphlet’s message was judgmental or shaming, and in particular the “gauge” for rating how serious the presenting child’s illness or injury was. The possibility of such an interpretation was noted when the initiative was launched, and to mitigate the concern, physicians were instructed to focus the discussion on preventing future unnecessary visits, rather than debating the appropriateness of the current visit. Nevertheless, physicians still found the pamphlet’s message to be judgmental and were concerned that it could harm their relationship with the patient and caregivers.

*I really thought that they felt judged and they felt patronized and I really didn’t like how that changed our patient-physician relationship*.

[Physician 8]

*It’s that social interaction when you know the parents are worried and they’re feeling like they’re being judged, that’s what makes it difficult. Our encounter is meant to be therapeutic, trying to get a therapeutic relationship going in a short period of time and have some sense of trust and quality from the parents’ perspective, and I think it [the pamphlet] kind of undermines it*.

[Physician 20]

A related barrier to pamphlet use was fear of negative reactions from caregivers. This was due either to a negative experience delivering the message (“once bitten, twice shy”) or to fear of what might happen, rather than in response to a specific incident. Indeed, certain physicians specifically noted that they disliked and avoided conflict, and were therefore reluctant to use the pamphlet. Two physicians even feared the possibility of legal action from caregivers if the pamphlet’s message was misinterpreted and the caregivers decided to not seek care for their children when they should have.

*My concern was it was going to get parents’ backs up. I’m there to educate, of course, but the last thing I need is for them to leave that room angry at me when I just tried to give them information that was relevant and actually useful. … It just felt like an added opportunity for them to get upset*.

[Physician 20]

Finally, a few physicians reported reaching for the pamphlet when they were particularly frustrated or annoyed by what seemed to them like a completely unnecessary visit. Giving the pamphlet to those caregivers allowed the physicians to channel their frustration productively and gave them a sense of having done something tangible to address what they perceived as waste.

*Things that would enable me to give it are when I just shake my head and say that was a complete waste of health care dollars …: this was a kid who stubbed their toe and there was no reason for them to be seen. It feels a bit cathartic to be able to give them the form, and in a nice way say “you didn’t need to be here”*.

[Physician 13]

## Discussion

Organizations often experience difficulties in implementing new initiatives and ensuring their sustainability, and hospitals are no exception. The landscape is littered with well-designed programs that addressed an important problem and had a good level of support at their inception, only to fail to gain traction and ultimately fade away, thus the need for studies of this nature that analyze the views of those involved in implementation. In terms of the “Choosing Wisely” educational initiative examined here, an important feature was the inclusion of PED physicians in the interdisciplinary design team. However, this proved to be insufficient to ensure the initiative’s success, in part because it is not always possible to anticipate all barriers during the design phase. Instead, additional efforts should have been made to solicit feedback and adjust the initiative during the initial roll-out. This is especially true since physicians can be a challenging group to bring on board when processes and practices are changed because, as professionals, they exercise a high degree of independence, and implementation strategies need to engage them in ways that are compatible with how they view their professional role [35]. Thus, clear and consistent support from organizational leadership throughout the implementation is essential; indeed, one of the barriers identified by the physicians that contributed to the failure of the “Choosing Wisely” initiative was a lack of clarity and inconsistent messaging from the hospital administration.

As compared to other educational initiatives to reduce low-acuity visits to the PED, the “Choosing Wisely” initiative was distinctive in its use of PED physicians to deliver the message to caregivers. In our review of educational programs to reduce low-acuity PED visits, we were unable to find any studies in which PED physicians were the primary educators; instead, that role was typically carried out by nurses, trained educators, clerical staff or the researchers themselves (see, for example, [18–24]). A meta-analysis examining the relationship between physician communication and patient adherence to treatment found a 19% higher risk of non-adherence among patients whose physician communicates poorly as compared to patients whose physician communicates well; furthermore, training physicians to communicate better enhances their patients’ adherence [25]. Thus, effective physician communication has a significant positive effect on changing patient behavior and supports the importance of engaging physicians in educational programs to help ensure the programs’ success.

Our analysis revealed 19 barriers and enablers that influenced physician participation in the “Choosing Wisely” educational initiative, which mapped to five TDF domains (Table 1 and Fig 1). The TDF provides a theory-informed approach to identify behavior determinants [33], from which implementation strategies can be developed to improve participation in, and sustainability of an initiative such as the one studied here. For example, an important barrier relating to beliefs about consequences was that physicians were uncertain as to the effectiveness of the initiative. While there was a drop in PED visits after the initiative was launched, there was no formal performance measurement strategy as part of the initiative. A performance measurement strategy is an important element of any initiative to reduce low-acuity PED usage, as it can help inform program design and implementation. Moreover, it can be used as a tool to create momentum for implementation of the initiative when the results are positive. Researchers have noted the importance of early wins in mobilizing participants and in consolidating efforts [36,37]. In the case of the “Choosing Wisely” initiative, daily or at least weekly communication about the numbers of pamphlets distributed, examples of positive interactions with caregivers and adjustments made in response to negative ones, along with trends in patient visit numbers, may have bridged the gap to more formal performance measurement. However, even if there had been such a strategy, the initiative’s short duration meant it would have been difficult to determine whether or not it was successful in changing caregivers’ behavior and reducing future low-acuity PED visits.

Another key domain influencing physician participation was emotion, manifested as fear of negative reaction from caregivers and the perception among physicians that the education message was judgmental or shaming. The rating of the current visit as part of the “Choosing Wisely” message opens the possibility for patient and family interpretation of the message as being judgmental, which was identified by physicians as a barrier for delivery of the message. General educational information, on the other hand, depersonalizes the issue as the message is not specifically targeted or customized to the patient and his/her family visit. Thus, physician participation could be improved by focusing the pamphlet’s message on the general educational component and downplaying or removing the assessment of the current visit’s acuity, or indeed anything that might be considered judgmental or elicit a negative reaction from the caregivers. In addition, while PED physicians were encouraged to use the pamphlet, it was left to their discretion and not framed as part of the “standard of care”. As a result, the pamphlet was used inconsistently, potentially creating the perception of singling out and judging some caregivers. Consistent use of the pamphlet with all patients would help avoid such perception.

An interesting observation was how physicians’ level of experience impacted their view of the initiative. Specifically, more experienced physicians were less likely to assume that all low-acuity PED visits should be diverted to other settings, and they were more likely to perceive changing caregivers’ behavior as being an intractable problem. This may result from the physicians having experienced other unsuccessful attempts to address the problem. On the other hand, physicians with less experience reported that the pamphlet was useful in that it added structure to their discharge instructions, and they tended to be more receptive to the training they received in using the pamphlet. Thus, a successful implementation strategy needs to tailor the program and its implementation appropriately to physicians according to their level of experience. “Different strokes for different folks” may be a more effective way of persuading a diverse group of physicians to embrace the initiative [38].

### Limitations of study

This initiative was implemented at a single children’s academic hospital in a large Canadian urban area, limiting its generalizability. However, we believe that the high-level conclusions derived from the study are relevant and transferable [30] to other settings that may wish to undertake a similar endeavor to reduce low-acuity PED visits. With regard to data collection, a significant number of physicians (23/42, or 55%) participated in the study; however, we have no information on the 19 physicians who declined to be interviewed. Thus, we cannot conclude with certainty that our sample is truly representative of all physicians; nonetheless, the barriers and enablers we identified are relevant to the majority of physicians involved. In addition, the absence of a performance measurement strategy meant we were unable to determine whether the program had an impact in reducing low-acuity visits to the PED. Finally, because this study was conducted after the initiative had been discontinued, we were unable to validate the physicians’ perceptions by interviewing caregivers after they received the message to determine (among other things) if they found the message judgmental, whether the message resonated with them and was retained, and whether it would or did change future behavior.

## Conclusions

PED physicians can play an important role in educating caregivers about appropriate PED use, but they are rarely involved in formal educational initiatives. We found that PED physicians were motivated to participate in the “Choosing Wisely” initiative because of the many problems created by high volumes of low-acuity PED visits. However, sustaining physician participation was a challenge. The barriers and enablers of PED physician participation that we identified mapped to five TDF domains: social/professional role and identity; beliefs about consequences; environmental context and resources; social influences; and emotions. These domains and associated barriers and enablers represent key factors influencing physician behavior towards the “Choosing Wisely” initiative and can help inform which behavior change techniques should be considered as part of the initiative’s design and implementation to improve physician participation. More broadly, the results of this study can also guide others who wish to develop similar educational initiatives that depend on physician participation, to help ensure their successful implementation.

## Supporting information

S1 Fig Annual visits to the CHEO PED by acuity. (PDF)

S2 Fig Weekly average of daily patient arrivals to the CHEO PED, 2013 to 2015. (PDF)

S1 File Choosing Wisely pamphlet. (PDF)
