# Supplementary figures and images for: Barriers and enablers to a physician-delivered educational initiative to reduce low-acuity visits to the pediatric emergency department
Source: PLoS One. 2018 May 29;13(5):e0198181. doi: 10.1371/journal.pone.0198181 (PMC5973597; doi:10.1371/journal.pone.0198181)

**S1 Figure:** Annual visits to the CHEO PED by acuity.

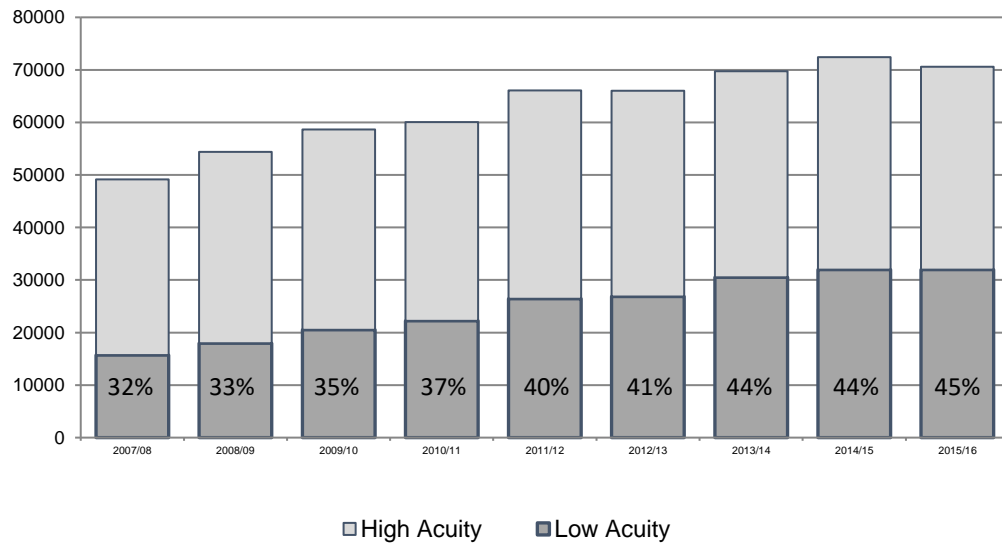

Supplement: S1 Fig — (PDF) [file pone.0198181.s001.pdf]

**S2 Figure:** Weekly average of daily patient arrivals to the CHEO PED, 2013 to 2015.

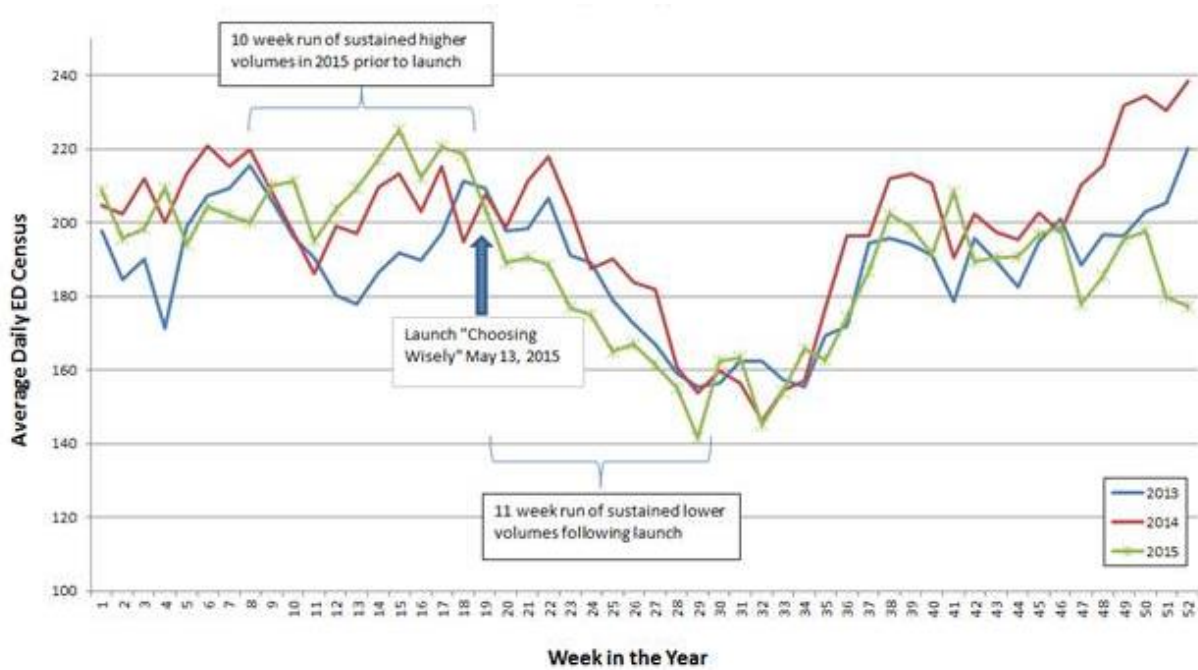

Supplement: S2 Fig — (PDF) [file pone.0198181.s002.pdf]
