# Supplementary material for: Barriers and enablers to a physician-delivered educational initiative to reduce low-acuity visits to the pediatric emergency department
Source: PLoS One. 2018 May 29;13(5):e0198181. doi: 10.1371/journal.pone.0198181 (PMC5973597; doi:10.1371/journal.pone.0198181)
Supplement: S1 File — (PDF) [file pone.0198181.s003.pdf]

We help children and families be their healthiest

Choosing Wisely

Good news!

Your child or teen is well enough to return home, where you can keep managing today's health problem. We were happy to help - it's our job and our passion. But sometimes, it's hard to provide the best care possible when families come to the Emergency Department when it's not really needed. We need your help to get this message out.

We get it-it's not always easy to decide if your child really needs to come to the Emergency Department.

But emergency department care costs the health care system a lot more than care in a family doctor's office or walk-in clinic -more than 10 times more! And it's harder for us to care for children and youth with more serious illnesses and injuries when we must also care for patients who could easily and safely be cared for in an office or clinic. **We need to keep the Emergency Department for emergencies.** Family doctors know their patients best, and have the skill and resources to manage most minor problems that crop up. Make sure you talk to your family doctor about these options.

Before you leave today, let's make sure you have answers to the following questions:

1. What was I most worried about today? If it happens again, how will I recognize when it might be more serious?
2. What symptoms were most distressing to my child or teen (fever, pain, etc.)? Do I now know how to relieve these symptoms at home while waiting to see my family doctor?
3. What questions do I still have? Do I know where to get more information about my child or teen's health?

Here's how serious the illness or injury was today...

The most seriously ill or injured patients receive care first, and are the reason we have emergency departments. Patients with less serious problems will wait longer. The gauge below will give you an idea of how serious your child or teen's problem was today, compared to other patients' needs. This will help you make your decision in the future.

Urgent
Most patients need emergency care that day.

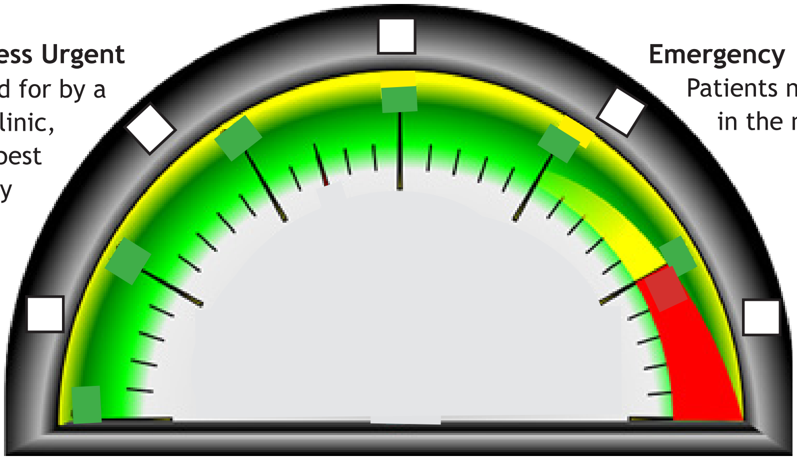

Less Urgent
Most patients can be cared for by a family doctor or walk-in clinic, though some injuries are best managed in the emergency department.

Emergency
Patients need assessment and care in the next 30-60 minutes.

Non-Urgent
Patients do not need care in the Emergency Department. Care can safely be given by a family doctor or walk-in clinic.

Life threatening
Patients need help right away. All physicians and staff will drop what they are doing to care for these patients.

Does my child need emergency care?

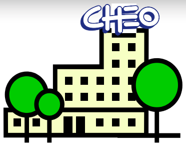

Here are some examples of when children need emergency care, and when they do not.
Please refer to the fact sheet we've given you about your child's condition for more information.

Emergency

Bring your child or teen to the **emergency department** for:

- **Fever** - less than 3 months old; immune system problems or complex chronic health problems; very sleepy or difficult to wake.
- **Breathing Problems** - respiratory distress (working hard to breathe; breathing faster than normal); pale skin, whitish or blue lips; asthma or wheezing not responding to usual puffers; or chest pain.
- **Vomiting or Diarrhea** - less than 3 months old; repeated vomiting and unable to keep liquids down (any age, if it lasts 8 hours or more); vomiting or diarrhea containing large amounts of blood; dehydration with dry mouth or no urine more than 8 hours.
- **Injuries** - head injury with loss of consciousness (passing out) or confusion or repeated vomiting; cuts that may need stitches; burns that blister and are larger than a Loonie; injury to arm or leg causing large swelling or inability to use the limb; eye injuries; or injury causing chest or stomach pain.
- **Rashes** - rash with fever that looks like tiny or expanding bruises.

Not an emergency

Bring your child or teen to your **family doctor** or a **walk-in clinic** for:

- **Fever** - healthy and vaccinated babies and children who appear generally well and playful when the fever is down with ibuprofen (Advil®, Motrin®) or acetaminophen (Tylenol®,Tempra®).
- **Breathing Problems** - nasal congestion and cough, even if it interrupts sleep; symptoms of the 'common cold'; mild asthma or wheezing that responds to usual puffers.
- **Vomiting or Diarrhea** - vomiting or diarrhea less than 3-4 times a day; ongoing diarrhea after 'stomach flu' (this can last for up to 2 weeks).
- **Injuries** - minor head injuries with no loss of consciousness, no confusion and no vomiting; scrapes and bruises where the injured part can still be used; sun burn.
- **Rashes** - recurring rashes or skin problems; rashes with cough and cold symptoms if the child looks well; mild hives that respond to antihistamines (Benadryl®) without difficulty breathing or throat/ tongue swelling.

Alternatives to Emergency:

1. **Call your family doctor or pediatrician.** Even if the office is closed, listen to the voice message for instructions and details about accessing care outside of office hours. Many family doctors have same-day, next day or walk-in hours during evenings and weekends. They may also offer 24/7 telephone advice to help you manage until they can see you.

2. **Visit a walk-in clinic.** In Ontario, call 211 (24 hours a day, 7 days a week) for information about walk-in clinics close to you. Or visit 211Ontario.ca and use the search feature. In Gatineau (starting May 15, 2015), call or visit the Mini-Urgence clinic, 500, Boul.de l'Hôpital, local 102. 819-966-6388

Looking for a family doctor?

In Ontario: Health Care Connect is a program that connects patients with primary care providers. Patients may register by phone or online. You will then be linked with a Nurse Care Connector who will help you find a doctor.

1. Call 1-800-445-1822 (Monday to Friday, 9:00am to 5:00pm). You will speak to a representative who will ask you a few questions about your location and specific needs.

2. On line at www.health.gov.on.ca/en/ms/healthcareconnect/public.

In Quebec: Please call your local CLSC, Info-Santé (811) or register online: www.csssgatineau.qc.ca. You may also contact the Collège des médicins du Québec at 1-888-633-3246 or www.cmq.org

Information you can trust

- CHEO www.cheo.on.ca (Programs & Health Info > A-Z listing)
- Canadian Pediatric Society www.caringforkids.cps.ca
- Hospital for Sick Children www.aboutkidshealth.ca
- Ottawa Public Health http://ottawa.ca/health www.parentinginottawa.com
- Mental health and addictions information and services www.ementalhealth.ca
- Info-Santé http://sante.gouv.qc.ca/en/systeme-sante-en-bref/info-sante-8-1-1/
